# Primary angioembolization in liver trauma: major hepatic necrosis as a severe complication of a minimally invasive treatment—a narrative review

**DOI:** 10.1007/s13304-022-01372-9

**Published:** 2022-09-04

**Authors:** Edoardo Segalini, Alessia Morello, Giovanni Leati, Salomone Di Saverio, Paolo Aseni

**Affiliations:** 1 Department of General and Emergency Surgery, ASST Ospedale Maggiore, Crema, CR Italy; 2 Department of Interventional Radiology, ASST Ospedale Maggiore, Crema, CR Italy; 3 Department of General Surgery, Ospedale Civile “Madonna del Soccorso”, San Benedetto del Tronto, AP Italy; 4 Department of Emergency, ASST Grande Ospedale Metropolitano Niguarda, Milan, Italy

**Keywords:** Major hepatic necrosis, Liver trauma, Angioembolization, Non-Operative Management

## Abstract

The liver is the second most commonly solid organ injured in blunt abdominal trauma. Liver injuries are classified according to the American Association for the Surgery of Trauma Injury Scale. The choice of Non-Operative Management is based on generalized clinical patients’ conditions combined with the evidence on CT scan imaging. To date, there are no consensus guidelines on appropriate patient selection criteria for those who would benefit from angiography and angioembolization. Major hepatic necrosis is a clinical condition of extended liver damage and is the most common complication after angioembolization. Large amounts of necrotic liver require therapy, but it is unclear if the better technique is debridements supplemented by percutaneous drainage procedures or definitive resection. A systematic review of the literature was performed with a computerized search in a database such as Medline for published papers on the use of angioembolization in trauma patients with hepatic injuries and on the most common complication, the major hepatic necrosis. The systematic review was conducted according to the recommendations of the 2020 updated Preferred Reporting Items for Systematic reviews and Meta-Analyses (PRISMA) guidelines. A total of 3643 patients were included in the study, suffering liver trauma and 1703 (47%) were treated with Non-Operative Management; angioembolization was performed 10% of cases with a variable rate between 2 and 20%. Patients developed different complications. Hepatic necrosis accounted for 16% ranging from 0 to 42%. 74% of patients underwent operative management with a mortality rate of 11%. High-grade liver injuries pose significant challenges to surgeons who care for trauma patients. Many patients can be successfully managed nonoperatively. In hemodynamically stable patients with arterial blush, without other lesions requiring immediate surgery, selective and super-selective AE of the hepatic artery branches is an effective technique. However, these therapies are not without complications and major hepatic necrosis is the most common complication in high-grade injures. Level III, Systematic review

## Introduction

In the past 30 years, Non-Operative Management (NOM) of solid organ injuries has become the standard of care for blunt trauma. The first angioembolization (AE) procedures were used for pediatric splenic trauma [1] and now AE represents the main treatment for solid organ injuries in stable adult patients. AE is an essential tool of NOM and it increases the success rate, avoiding invasive surgical procedures, especially in abdominal trauma, such as in liver injuries.

The second most commonly involved solid organ (after spleen) to be injured in blunt abdominal trauma is the liver because of its location and its relationship with other structures in the abdomen [2] and liver injury is the most common cause of death in such trauma with a 10–15% mortality rate [3].

Liver injuries are classified according to the American Association for the Surgery of Trauma (AAST) Injury Scale; any injury in the presence of a liver vascular injury or active bleeding contained within liver parenchyma defines a grade III [4]. Regardless of this classification, the choice of NOM (Non-Operative Management) rather than OM (Operative Management) is based on generalized clinical patients’ conditions combined with the evidence on CT scan imaging. NOM includes the “watch and wait” strategy and the AE procedure of branches of the hepatic artery. In the last decades, diagnostic angiography and eventually therapeutic AE grow up as an essential component in a successful NOM in patients with arterial bleeding, as demonstrated by radiologic imaging of contrast extravasation, also referred to as “contrast blush”.

Although liver trauma is still one of the leading causes of death after blunt abdominal injuries, total death rates and liver-related deaths decreased in the last decades with an increase in the total number of injuries due to the population growth and the rise in total trauma volume.

Major hepatic necrosis (MNH) is a clinical condition of extended liver damage which involves more than 75% of the liver and is the most common complication after AE; it can be also a localized ischemic injury or a more generalized severe injury with submassive hepatic necrosis involving 26–75% of the parenchymal volume [5]. The liver has a high resistance to ischemic injuries due to its double vascularization, the arterial and the portal venous system. However, after major trauma with high-grade liver injuries (grade IV–V ASST), there is a massive hepatic tissue and major biliary or vascular structures disruption with a compromised venous portal flow that is no longer sufficient to maintain the tissue viability [6, 7]. When complications develop in patients with MHN, it means that they require significantly more blood transfusions and have a significantly longer length of stay; these patients are more likely to have undergone operative management.

While the increasing use of NOM yielded a significant decrease in the overall mortality, the prognosis of haemodynamically unstable patients with complex (grade IV–V AAST) liver injury is still poor as their treatment and decision-making process are challenging for the trauma surgeon.

Small amounts of the necrotic liver may be managed expectantly, large amounts of necrotic liver require therapy, but it is unclear if the better technique is a series of multiple debridements supplemented by percutaneous drainage procedures or definitive resection. In case of demonstrated MHN with evidence of superinfection of the necrotic liver parenchyma, prompt surgical management is indicated.

Currently, retrospective case series constitute the majority of investigations on the use of nonoperative management in hepatic injury without comparative groups and with no standardization for patient selection or reporting.

## Methods

### Search strategy

A systematic review of the literature was performed with a computerized search in a database such as Medline for published papers on the use of AE in trauma patients with hepatic injuries and on the most common complication after AE, the MHN. The primary search strategy was to check liver, hepatic, trauma, adult, hemodynamic stability, nonoperative, conservative management, AE, complications, major hepatic necrosis combined with the Boolean operators AND/OR. Prior to the search, inclusion, and exclusion criteria were defined. The inclusion criteria were the study population consisted of patients with hepatic bleeding from traumatic cause, treated with AE as primary intervention, papers treating the development of complications after primary AE. The exclusion criteria were case reports, case series with fewer than five consecutive patients, AE performed after operative management (for example, hepatic packing), papers describing the treatment of hepatic hemorrhage not due to traumatic injuries.

The primary outcome was the efficacy rate of AE in obtaining control of arterial hepatic hemorrhage and the development of hepatic complications such as major hepatic necrosis. Secondary outcomes of interest included treatment of the MHN, mortality rate, and liver-related mortality rate. Search results were limited to humans, English language, and papers published after 1990.

### Data extraction and synthesis

Two reviewers [PA, AM] independently scrutinized the titles and abstracts of the papers retrieved to identify relevant articles for data extraction, resolving discordances by mutual discussion. Then, the authors scrutinized the full length of the remaining papers for eligibility criteria and at least found 11 retrospective studies published between 2002 and 2018.

The data extracted included the year of publication, study type, study time period, indication for AE, the number of patients treated with AE, complications after AE included major hepatic necrosis (MHN), treatment for MHN, mortality, and liver-related death.

**Fig. 1:**
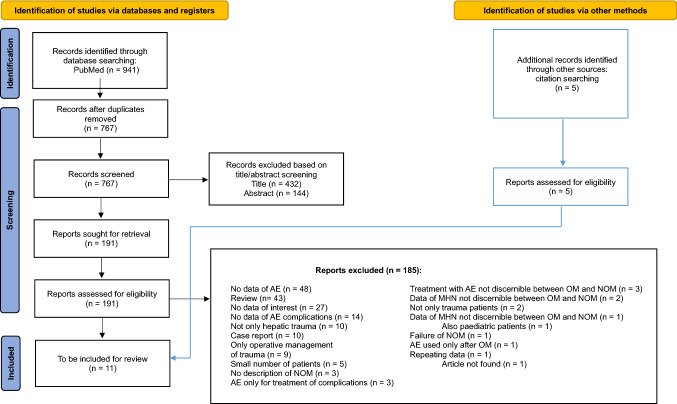
Search results and selection of included studies

This systematic review was conducted according to the recommendations of the 2020 updated Preferred Reporting Items for Systematic reviews and Meta-Analyses (PRISMA) guidelines [8] (Fig. 1).

## Results

After the screening process had been completed, 11 articles were considered suitable for the study. All the articles were a retrospective study, published between 2002 and 2018, and two were multicentre (Table 1).

**Table 1:** Characteristics of the included studies

References	Date of publication	Years	Design	Inclusion criteria	Exclusion criteria	Total sample size
Dabbs et al. [9]	2009	2002–2007	Retrospective Single center	Hepatic trauma AE as initial management	–	538
Kong et al. [10]	2014	2002–2011	Retrospective Single center	Blunt liver injuries Selective AE Hemodynamic stability or stabilized	Penetrating injuries Hemodynamically unstable patients and/or unstable hemodynamics during NOM	–
Letoublon et al. [11]	2011	1999–2008	Retrospective Single center	Nonpenetrating liver injuries Hemodynamic stability or stabilized	Hemodynamic unstability	183
Li et al. [12]	2014	2007–2012	Retrospective Single center	Liver trauma Hemodynamic stability	Hemodynamic unstability	268
Misselbeck et al. [7]	2009	1997–2005	Retrospective Single center	Liver injuries	–	707
Mohr et al.	2003	1995–2002	Retrospective Single center	Hepatic injury	–	866
Monnin et al.	2007	2000–2005	Retrospective Single center	Blunt hepatic trauma	–	84
Saltzherr et al.	2010	1995–2001 2002–2008	Retrospective Single center	Liver injuries	Initially treated at other hospitals	116
Sekine et al.	2018	2000–2010	Retrospective Multicenter	Blunt hepatic injuries	–	72
Wahl et al. [13]	2002	1997–2001	Retrospective Multicenter	Blunt hepatic injuries	–	126
Xu et al. [14]	2017	1998–2015	Retrospective Single center	Blunt hepatic trauma	Patients who died shortly after arriving at the hospital Penetrating trauma Iatrogenic misadventures	683
TOT						3643

A total of 3643 patients were included in the study, all of these have suffered liver trauma and 1703 (47%) were treated with NOM. Hemodynamic stability with evidence of active hepatic extravasation on abdominal CT was the main indication for angiography and AE. Patients treated with pAE after hepatic trauma was 10% (364 pAE on 3643 total hepatic trauma); the rates were very variable, ranging from 2 to 20%.

From every paper, we were able to pull data about the grade of hepatic injury that suffered patients undergoing AE: the average injury grade score is 3.48 with a median of 4; in fact, 143 patients (39% of total AE) had hepatic injury of grade II or III, 117 (32%) had hepatic injury of grade IV and 41 (11%) of grade V or VI (Table 2).

**Table 2:** Type of management and complications

References	Total sample size	Sample size NOM	Sample size pAE *N* (% of total)	Grade injury *N* (% of total AE)	Hepatic necrosis *N* (% of total pAE)	Grade injury *N* (% of total HN)
Dabbs et al. [9]	538	116 (22%)	71 (13%)	Gr III 16 (23%) Gr IV 44 (62%) Gr V 11 (15%) Gr VI 0 (0%)	30 (42%)	Gr III 2 (6%) Gr IV 20 (67%) Gr V 8 (27%)
Kong et al. [10]	–	–	70	Gr II 13 (18%) Gr III 25 (36%) Gr IV 23 (33%) Gr V 9 (13%)	11 (16%)	–
Letoublon et al. [11]	183	151 (83%)	13 (7%)	Gr III 6 (46%) Gr IV 7 (54%)	1 (8%)	–
Li et al. [12]	268	72 (27%)	22 (8%)	Gr II 1 (4%) Gr III 5 (23%) Gr IV 12 (55%) Gr V 4 (18%)	0	–
Misselbeck et al. [7]	707	58 (8%)	20 (3%)	> 80% grade III or more	4 (20%)	Gr III 1 (25%) Gr IV 3 (75%)
Mohr et al	866	736 (85%)	11 (1%)	–	3 (27%)	Gr IV 3 (100%)
Monnin et al.	84	–	10 (12%)	Gr III 2 (20%) Gr IV 7 (70%) Gr V 1 (10%)	1 (10%)	Gr V 1 (100%)
Saltzherr et al.	116	84 (72%)	23 (20%)	Gr I–II 7 (31%) Gr III 9 (39%) Gr IV 6 (26%) Gr V 1 (4%)	3 (13%)	Gr V 1 (33%)
Sekine et al.	72	35 (49%)	14 (19%)	Gr V 3 (21%)	1 (7%)	–
Wahl et al. [13]	126	100 (79%)	6 (4 successful, 3%)	Gr III 3 (50%) Gr IV 3 (50%)	0	–
Xu et al. [14]	683	351 (51%)	114 (106 successful, 16%)	Gr II 34 (30%) Gr III 32 (28%) Gr IV 15 (13%) Gr V 11 (10%) Gr VI 1 (1%)	3 (3%)	–
TOT	3643	1703 (47%)	364 (10%)	Gr I / Gr II 55 (15%) Gr III 88 (24%) Gr IV 117 (32%) Gr V 40 (11%) Gr VI 1 (0.3%)	57 (16%)	Gr I / Gr II / Gr III 3 (5%) Gr IV 26 (46%) Gr V 10 (18%)

### AE and hepatic necrosis

After pAE, patients developed different complications. Hepatic necrosis accounted for 16% (57 cases on 364 pAE) and a range from 0% of Li (0 cases on 22 procedures) and Whal (0 cases on 4 procedures) to 42% of Dabbs (30 cases on 71 procedures) [12, 13].

Only in five studies, a correlation was found between hepatic necrosis and the grade of the hepatic trauma. In Dabbs et al. [9], 67% (20 patients) had a hepatic trauma classified as grade IV, 27% (8 patients) a grade V and 6% (2 patients) a grade III. In Misselback et al., 75% (3 patients) had a hepatic trauma classified as grade IV, 25% (1 patient) a grade III. In Mohr et al. [15], a lesion of grade IV was reported in all patients that developed hepatic necrosis. Meanwhile, in Monnin et al. and Saltzherr et al., the only case recorded was a grade V (Table 2).

### Treatment and mortality

Fifty-seven patients on 364 treated with primary AE developed hepatic necrosis (16%) after primary AE, and the diagnosis was confirmed with a contrast CT scan. Fifteen cases (26% of hepatic necrosis) were treated conservatively, and of these, only one patient died of multiple organs failure. Forty-two cases (74% of hepatic necrosis) underwent operative management: 18 hepatic resections or lobectomy and 24 multiple debridements. The mortality rate was 11% (6 cases on 57 total hepatic necrosis): 3 patients who underwent hepatic resection died for massive hemorrhage during the operation, multiple organ failure, and sepsis; 2 patients died for sepsis and multiple organ failure following multiple debridement and necrosectomy. Only one patient died after conservative management with only AE (Table 3).

**Table 3:** Complications’ treatment and mortality

References	Total sample size	Sample size pAE *N* (% of total)	Hepatic necrosis *N* (% of total pAE)	HN treatment *N* (% of total HN)			Deaths related to HN after treatment *N* (% of total HN)		
				NOM	Resection	Debridement	NOM	Resection	Debridement
Dabbs et al. [9]	538	71 (13%)	30 (42%)	0	16 (53%)	14 (47%)	–	2 (7%)	0
Kong et al. [10]	–	70	11 (16%)	11 (100%)	0	0	0	–	–
Letoublon et al. [11]	183	13 (7%)	1 (8%)	0	0	1 (100%)	\	\	1 (100%)
Li et al. [12]	268	22 (8%)	0	–	–	–	–	–	–
Misselbeck et al. [7]	707	20 (3%)	4 (20%)	1 (25%)	0	3 (75%)	1 (25%)	–	0
Mohr et al. [15]	866	11 (1%)	3 (27%)	0	1 (33%)	2 (67%)	–	1 (33%)	0
Monnin et al.	84	10 (12%)	1 (10%)	0	0	1	–	–	1 (100%)
Saltzherr et al.	116	23 (20%)	3 (13%)	3	0	0	0	–	–
Sekine et al.	72	14 (19%)	1 (7%)	0	1	0	–	0	–
Wahl et al. [13]	126	6 (4 successful, 3%)	0	–	–	–	–	–	–
Xu et al. [14]	683	114 (106 successful, 16%)	3 (3%)	0	0	3	–	–	0
TOT	3643	364 (10%)	57 (16%)	15 (26%)	18 (32%)	24 (42%)	1 (2%)	3 (5%)	2 (4%)

In this review, we focused on patients handled primarily with AE, and we excluded patients managed with an operative treatment such as laparotomy and hepatic packing and secondarily with AE.

## Discussion

The first classification of solid organ injuries after trauma uniformly adopted was published in 1989 by Moore et al. and updated in 1994; it was organized like an Organ Injury Scale based on the anatomic description of every injured type of lesions in solid organs. The severity of liver injuries was ranked from 1 to 6, representing grade 1 the least and grade 6 the most severe injury classified as a destructive lesion incompatible with survival [16, 17]. In 2018, Kozar et al. reviewed the solid organ injury scale for spleen, liver and kidney and introduced three sets of criteria to assign a grade (imaging, operative and pathologic) to describe better and compare equivalent injuries treated in one fashion versus another. The final AAST grade is attributed by the highest grade assessment obtained by the evaluation of the three sets of criteria. When there is more than one liver injury, the higher grade of injury is used to classify them [4]. According to 2018 AAST Liver Injury Scale, any injury in the presence of a liver vascular injury or active bleeding contained within liver parenchyma represents a grade III injury. In the 1994 injury scale, there was not the specification about active bleeding, thus in some cases AE was employed even in grade I and II injuries, such as reported in Kong, Li, Saltzherr and Xu [10, 12, 14]. In these reviews, the authors reported that AE was performed in case of active bleeding, as a CT finding, within parenchymal laceration or hepatic hematoma and this condition defined the grade injury.

Contrast CT scan is the fundamental diagnostic tool for the primary approach to identify active bleeding that can cause a hemorrhagic shock if left untreated; it can also reveal a portosystemic shunt or a portal thrombosis. Toguchi et al. report the case of a patient with liver trauma that underwent unenhanced computed tomography of the abdomen. Additional damages have not been recognized with this diagnostic investigation, and after the worsening of her clinical conditions, a contrast-enhanced CT revealed lacerations of the liver, hemoperitoneum, arterial contrast blush, and portal-systemic venous shunt. After hepatic trauma can be multiple injuries that remain unrecognized if performing an unenhanced CT scan [18]. CT scan also has an essential role in identifying necrotic complications: some authors recommend performing it only in case of clinical deterioration during the follow-up period after the AE procedure.

The WSES guidelines for liver trauma (2016), Kaptanoglu et al. (2017), and the evidence-based guidelines for NOM (2017) concluded that the AAST grade estimated by CT scan is not enough to determine the optimal treatment in abdominal trauma. The anatomical description is fundamental but must be integrated with the hemodynamical condition and associated injuries. Patients with all grade liver injury, hemodynamically stable or stabilized by initial resuscitation, without signs of peritonitis or associated abdominal lesions that require immediate surgical exploration, may be managed with NOM [19, 20, 21].

To date, there are no consensus guidelines on appropriate patient selection criteria for those who would benefit from angiography and angioembolization (AE). Contrast-enhanced computed tomography (CT) has been shown to identify those at risk for impending failure of Non-Operative Management: intraperitoneal contrast extravasation and hemoperitoneum in six compartments on CT scan both indicate massive or active hemorrhage; these findings pose patients at high risk for the need of operation also if hemodynamically stable [22].

AE becomes the first treatment in hemodynamically stable patients with a success rate of 87% for Samuels et al. [23] and a rate ranging from 80 to 97% for Virdis and colleagues [24].

Evidence of decrease in mortality rate is described by Suen et al. that analyzed three periods of 5 years each from 1999 to 2013 and compared them: the overall mortality decreased from 21.1 to 7.7%, and these numbers decreased from 18.8 to 3.6% if considered only patients who died within 24 h of arrival at the hospital [25].

Two recent reviews show a death rate of 9.6% (range 0–27%) in patients undergoing NOM with AE and a total liver-related death rate of 5.6% (range 0–19.2%) [6] and Virdis confirms the trend with five mortalities reported and a death rate of 0.7% (range 1–18%) [24]. However, AE is not without complications: rebleeding, liver necrosis and abscess, biliary leak and biloma, acute alithiasic cholecystitis, gallbladder necrosis, and abdominal compartment syndrome can follow and complicate the procedure. Liver necrosis and abscess, and gallbladder necrosis seem to be related to ischemia following AE [24].

The development of major hepatic necrosis (MHN) can be a severe ischemic complication related to the AE procedure in patients with liver trauma. This complication means that patients with MHN had higher grade injuries, required significantly more blood transfusions and had a significantly longer length of stay; these patients were more likely to have undergone operative management. Virdis et al. identify this complication in 8 studies with a mean incidence of 8.6% and a range between 4 and 45% [24]. Dabbs et al. highlight that there is a possible rise in ischemic liver complications during last years much more than in past years potentially related to the increased use of AE as the first treatment in patients with high-grade liver injuries. The overall complications rate after AE is about 60%, and the most frequent complications are bile leak, abscess, and MHN. MHN is becoming more common: it occurs in 42% of patients that undergo AE alone, in 65% of patients treated with combined AE and damage control laparotomy, and in 67% of patients treated with AE and liver packing [9]. All the authors concluded that MHN is a common clinical evolution that occurs mainly in patients with high-grade hepatic injury (grade IV–V–VI) according to the AAST classification. In these high-grade traumas, the disruption of the hepatic tissue and vascular structures is massive, and it requires a therapeutic arterial embolization of proximal branches or multiple distal branches with the effect of larger ischemic damage that may predispose to a greater devascularization and development of hepatic necrosis more frequently than in low-grade traumas. In addition, the association with portal thrombosis or thrombosis in any other branch of the hepatic vascular system can contribute to the final ischemic damage. Letoublon et al. [11] report three cases of hepatic necrosis: one of them occurs after a second angiography, performed for the failure of the pAE; in another case, they demonstrate thrombosis of the right branch of the hepatic artery despite a super-selective AE [11].

Many authors try to connect the development of this complication with the material and technique used during AE. Gel foam is the most commonly used in many studies (gelatin sponge particles and stainless-steel coils in Hagiwara; gel-foam and/or stainless-steel coils, polyvinyl alcohol particles in Mohr; gel-foam or intraarterial coiling in Misselbeck; gel-foam, coil, or both in Dabbs) [7, 15, 25, 26], but only two papers [7, 9] have related the necrosis with these materials, observing that complications occur very frequently after procedures with gel-foam; however, in the absence of a statistical significance, further evidence is needed.

Hepatobiliary complications are the natural evolution of this treatment, but their incidence can be reduced using selective or super-selective AE. Kong in 2014 reports a complication rate of 100% after intrahepatic branches and extrahepatic trunk embolization, meanwhile only an 11% rate after selective embolization, including 6% of patients after super-selective embolization [10].

In 2017, Xu and colleagues compared the two different techniques and found that 25.4% of patients were treated with a combined embolization of the extrahepatic trunk and intrahepatic branches (CEETIB); meanwhile, 74.6% underwent a simple selective embolization of the arterial branches (SSEAB), and in most of them a super-selective embolization of the terminal branches of the intrahepatic artery was employed. Complications like liver necrosis, gallbladder infarction, and hepatic abscess can be seen more often after a CEETIB. SSEAB enables to embolize only the injured tissue to permit the formation of clots and facilitate hemostasis, whereas the blood flow from the extrahepatic trunk continues to supply the rest of the liver. A study from Xu et al. seems to confirm that selective or super-selective embolization allows reducing the percentage and the severity of complications [14].

Although it is impossible to reduce the rate of this complication to zero, the development of selective and super-selective embolization techniques has allowed lowering its percentage and severity. Patients managed with AE must be monitored more frequently with daily clinical examination and blood tests. CT scan becomes paramount in case of worsening symptoms like abdominal pain with clinical signs such as tachycardia, fever, leukocytosis, elevated transaminases and hyperbilirubinemia, elevated lactate, and coagulopathy. These findings are very common in patients after major trauma, and clinicians must consider the possibility of MHN in the differential diagnosis of a worsening clinical scenario [9, 27].

The correct timing for treatment of MHN should discussed case by case, considering not only the AAST grade but, above all, the general clinical condition of the patients; in fact, it is not necessary to treat all patients with hepatic necrosis. As reported by Abdelrahaman et al., massive hepatic necrosis can be associated with a stable clinical state and can be treated conservatively (mainly in young patients); the liver can also heal thanks to its extraordinary regenerative properties [28]. However, if there is an operative indication, according to Dabbs et al. [9], delaying surgery is not associated with higher survival. Smaller areas of necrosis can be managed with antibiotics, but patients with sepsis and bacteremia need percutaneous drainage; if both fail, then open or laparoscopic technique needs to be considered for hepatic necrosectomy [21].

In case of infected necrosis or patients with necrosis and progressive worsening of the clinical condition, liver resection guarantees efficacy comparable to multiple debridements of necrotic liver tissue but is associated with longer operation time and greater bleeding [29].

Since there was no clear protocol for care, it is impossible to know why an individual surgeon selected lobectomy versus multiple debridements. The decision to proceed with lobectomy or hepatic resection versus debridement and percutaneous drainage is based on multiple factors, including the patient’s physiology, how amenable the lesion is to resection, the size and location of the area of necrosis, the experience of the surgeon, and the likelihood of success of each treatment algorithm [25].

The need for orthotopic liver transplantation (OLT) after liver trauma associated or not to AE is restricted. However, since the mortality rate of severe and complicated hepatic injuries remains significantly high, reaching 46% for grade IV and 80% for grade V hepatic injury, OLT must sometimes be considered the only rescue procedure available. Indications for OLT can be grouped in the following clinical setting: (a) uncontrollable massive bleeding requiring total vascular exclusion of the liver, (b) post-operative evolution toward acute liver failure following massive hepatic necrosis associated or not with an AE procedure, (c) major injuries of the portal vein and of the hepatic hilum that cannot be reconstructed [30].

A limitation regarding this review is that only a paper reported information about concomitant injuries to other organs [11] and Dabbs compared patients who developed MHN with those who did not and considered the ISS score which is based on the Abbreviated Injury Scale (AIS) and takes into account the three (out of six) most severe system injuries and, therefore, the single lesions are not specified. The considered papers do not specify whether the patients were hypotensive before the procedure; the hemodynamic status of patients before the AE remains unknown, thus it is not clear whether hepatic necrosis may be related to a prolonged hypotension before AE. When AE was performed, all patients were hemodynamically stable either because never unstable before or because they were responsive to initial resuscitation.

## Conclusion

High-grade liver injuries pose significant challenges to surgeons who care for trauma patients. Many patients can be successfully managed nonoperatively, but there are still patients that require laparotomy. AE seems a logical paradigm shift of damage control techniques to control hepatic hemorrhage. In hemodynamically stable patients with liver trauma and arterial blush on contrast CT scan, without other lesions requiring immediate surgery, selective and super-selective AE of the hepatic artery branches is an effective technique. However, given the nature and severity of these injuries, these therapies are not without complications. MHN is the most common complication in high-grade injures, often associated with other conditions such as the severity of trauma, hemodynamic instability, longer hospital stay, and higher transfusion requirements.

During the last years, AE has allowed to avoid complex surgical techniques and improve the success rate of the Non-Operative Management approach, reducing patient mortality. Careful clinical and laboratory follow-up after AE seems paramount to detect and treat this complication early. Risk assessment indicators for liver trauma should be developed in the future, and multicentre trials should provide more indications on the best therapeutic management by comparing different operative options and techniques to minimize the post-operative risk of major hepatic necrosis.
